# Causal deep learning to personalize medicine: Which intensive care patients with sepsis will benefit from corticosteroid therapy?

**DOI:** 10.1016/j.jointm.2025.07.002

**Published:** 2025-09-23

**Authors:** Ameet Jagesar, Louk Smalbil, Etienne Galea, Tristan Struja, Tariq Dam, Paul Hilders, Martijn Otten, Laurens Biesheuvel, Armand Girbes, Patrick Thoral, Mark Hoogendoorn, Paul Elbers

**Affiliations:** 1 Department of Intensive Care Medicine, Center for Critical Care Computational Intelligence (C4I), Amsterdam Medical Data Science (AMDS), Amsterdam Cardiovascular Science (ACS), Amsterdam Institute for Infection and Immunity (AII), Amsterdam Public Health (APH), Amsterdam UMC, Vrije Universiteit, Amsterdam, The Netherlands; 2 Quantitative Data Analytics Group, Department of Computer Science, Faculty of Science, Vrije Universiteit, Amsterdam, The Netherlands; 3 Medical University Clinic, Division of Endocrinology, Diabetes & Metabolism, Kantonsspital Aarau, Aarau, Switzerland

**Keywords:** Sepsis, Decision support systems, Causality, Artificial intelligence, Glucocorticoids

## Abstract

**Background:**

Sepsis, defined as life-threatening organ dysfunction due to dysregulated host response to an infection, often requiring intensive care treatment. There is a strong rationale for the administration of corticosteroids for immunomodulation; however, clinical trials are inconclusive, which may be attributable to heterogeneity in therapeutic effects between individual patients. Leveraging deep learning within a causality framework, we aimed to identify for which intensive care patients with sepsis corticosteroids lead to improved survival.

**Methods:**

We trained the treatment agnostic representation network (TARNet) to estimate the reduction in predicted probability of 28-day mortality following initiation of corticosteroid treatment of intensive care patients with sepsis. We used the freely available and public AmsterdamUMCdb ICU database for causal model development, considering 19 predictor variables from the first 24 h of admission, and validated the model with Medical Information Mart for Intensive Care (MIMIC-IV) version 2.2 data. A cut-off of 10% reduction in predicted probability of mortality was used to classify treatment responders.

**Results:**

According to the Sepsis-3 criteria, a total of 2920 admissions in AmsterdamUMCdb were eligible. Of these, 1378 were assigned to the intervention group and 1542 to the control group. Internal validation of predictions of the observed outcomes showed an area under the receiver operating characteristic curve (AUROC) of 0.79, while external validation yielded an AUROC of 0.71. Covariate balance of the TARNet model latent representation, as measured by the Wasserstein distance, was 3.6 × 10⁻⁷ for the internal data set and 4.2 × 10⁻⁷ for the external data set. Based on the estimated reduction of predicted mortality, a distinction was made between treatment responders (*n*=245), non-responders (*n*=2098), and those predicted to be harmed by corticosteroid treatment (*n*=577).

**Conclusions:**

Corticosteroid treatment responders were those with severe metabolic acidosis and impaired circulation, whereas patients who were less ill based on these parameters were more likely to have increased mortality rates by corticosteroid treatment. There was also a notable discrepancy between the model’s suggestions and the physicians’ treatment that was carried out, implying improvements in the clinical assessment of patients with sepsis are necessary. Given recent years have not yielded new treatments for sepsis, computational clinical decision-support systems are worth exploring.

## Introduction

Sepsis is a life-threatening condition characterized by organ dysfunction as a result of a dysregulated host response to infection. With decades of clinical and scientific progress, mortality due to sepsis is slowly declining but remains high, with an average 30-day survival rate of only 60%–70% for septic shock, even in developed countries.^[1]^ Patients with sepsis often require organ support in the intensive care unit (ICU), further underlining the clinical burden of the disease. Furthermore, those who survive frequently require intensive rehabilitation to regain functionality for daily activities.^[2]^ These major strains on hospital and rehabilitation resources make sepsis a large source of expenditure for many healthcare systems.^[3]^ The proportional increase in the older population is likely to exacerbate this as they are more susceptible to developing sepsis and having poorer outcomes.^[4]^ Early and appropriate treatment is crucial for alleviating the sepsis burden. Measures, including early administration of antibiotics and proper source control, are associated with a decrease in mortality.^[5^^,^^6]^

Another treatment modality is the use of corticosteroids aimed at inhibiting the severe systemic inflammatory response in sepsis. However, potential drawbacks include the risk of opportunistic infections due to inhibition of the immune system, possibly complicating the disease with a superinfection.^[7]^ A recent meta-analysis showed that corticosteroids might reduce mortality in adult patients with sepsis, regardless of the presence of shock.^[8]^ These insights are also addressed in the latest guidelines regarding sepsis treatment, which recommend administering corticosteroids to adult patients in septic shock.^[9]^ Nevertheless, these recommendations are based on low-certainty evidence and reflect the results of multiple inconclusive trials. The majority of the included trials have shown no statistically significant benefit in terms of reducing mortality, an endpoint that signifies a clear clinical benefit to the patient. This lack of significance can be attributed to the nature of clinical trials in the ICU, which are characterized by a heterogeneous population receiving a treatment that has a relatively small effect size for mortality. This holds especially true for sepsis, given that it can be subclassified based on numerous criteria such as primary infection, activated immune pathways, and severity of inflammation. In addition, the decreasing baseline mortality rates require larger sample sizes and associated resources to demonstrate statistical significance.^[10^^,^^11]^ These factors may cause many trials in the ICU to be underpowered and undermine incentives for future trials of adequate size.

Given that conventional trials have not been able to guide physicians towards optimal clinical decision-making for patients with sepsis, we propose that data-driven, personalized medicine could fill that gap. To achieve this goal, we leveraged the large scale and granular data provided by public ICU databases.^[12]^ Combining these databases with a state-of-the-art causal deep learning method, our objective was to identify patients with sepsis who respond to corticosteroid treatment with improved survival rates. We hypothesize that the most severely ill patients, based on physiological measurements, would benefit from corticosteroids, as suggested by previous literature.

## Methods

Access to the data sets was requested. Data extraction was performed by script (queries) of Standardized Query Language (SQL) in Google BigQuery. Data processing was performed using Python version 3.11, published by the Python Software Foundation. All code and package dependencies are available on the GitHub repository, below: https://github.com/ajagesar/causal_learning_for_sepsis_steroid_responders.

### Patients and preprocessing

The data for model development and testing were obtained from AmsterdamUMCdb, a freely accessible European database consisting of de-identified health data for 23,106 admissions in a Dutch tertiary ICU between 2003 and 2016.^[13]^ Admissions were selected based on the criteria of the Sepsis-3 definition.^[14^^,^^15]^ Admissions with missing sex data were excluded because these could not be imputed based on normal values. The following features were extracted: sex, age, weight, height, ventilation status, partial pressure of oxygen (PaO_2_) /fraction of inspired oxygen (FiO_2_), vasopressor dosage, pH, heart rate, blood urea nitrogen, creatinine, C-reactive protein, glucose, lactate, white blood cell count, temperature, and potassium, sodium, and bicarbonate levels. The most abnormal value within a 24-h window after admission was extracted as appropriate for the diagnosis sepsis. The highest values were selected for urea, creatinine, C-reactive protein, glucose, lactate, white blood cell count, potassium, sodium, and temperature. The lowest value was selected for bicarbonate. For heart rate, the median value within the 24-h window after admission was selected. The following missing variables were assumed to be Missing at Random: mechanical ventilation status, vasopressor dosage, and PaO_2_/FiO_2_. Absence of these values was assumed to indicate that these patients were not being mechanically ventilated, did not receive vasopressors, and had a normal PaO_2_/FiO_2_ (as this was no indication to measure it). These were then imputed with 0 (no treatment being given) or a value of 400 for PaO_2_/FiO_2_ to indicate a lack of oxygenation impairment. The other measurements were imputed with median values.

The outcome was 28-day mortality, defined as death within 28 days after ICU admission. The intervention was defined as the initiation of therapeutic dosages of corticosteroids within 72 h after admission, a binary treatment that was defined as >200 mg hydrocortisone or equivalent based on Daenen et al.^[18]^

For external validation, we used the Medical Information Mart for Intensive Care (MIMIC)-IV version 2.2.^[19]^ Patients with sepsis were also selected according to the Sepsis-3 definition. The features and outcome were preprocessed following the same steps as in the AmsterdamUMCdb database. The exhaustive list of features, criteria for extraction, imputation methods, and definition of interventions can be found in the Supplementary Materials.

### Model development and evaluation

**Figure 1:**
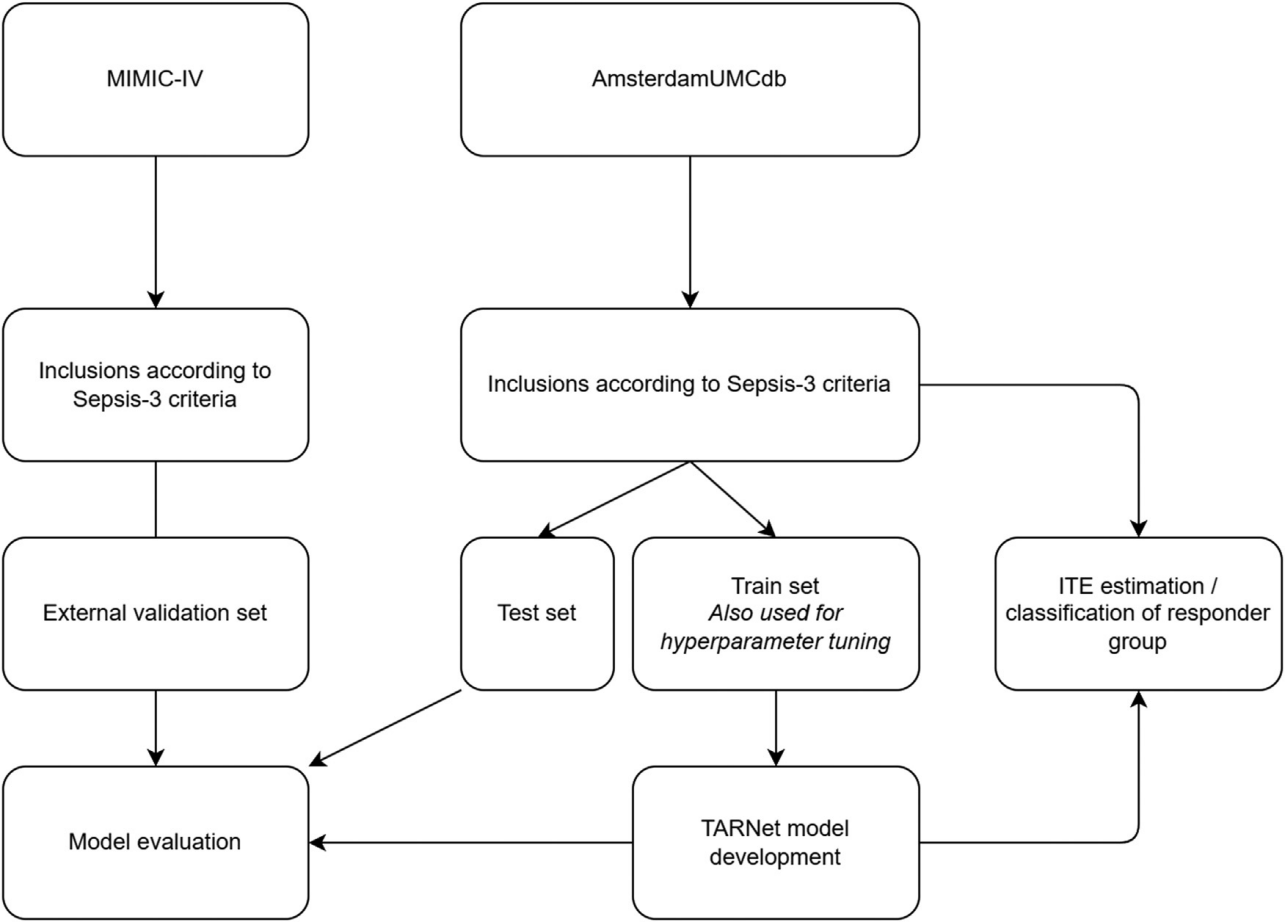
Schematic representation of the pipeline. ITE: Individual treatment effect; MIMIC-IV: Medical information mart for intensive care IV; TARNet: Treatment agnostic representation network.

A pipeline was built with the following steps: data splitting, data scaling, data imputation, feature ranking, hyperparameter tuning, model development, and model evaluation (Figure 1). The preprocessed data from AmsterdamUMCdb were split into a training (80%) and test (20%) set. The training set was scaled using min-max normalization. Ranking of all extracted variables by importance was performed using recursive feature elimination with cross-validation on the training set. Hyperparameter tuning was carried out using the Optuna library.^[20]^ The model for which hyperparameter tuning was performed was a treatment agnostic representation network (TARNet).^[21]^ Using 20% of the training set for cross-validation, the model was optimized for area under the receiver operating characteristic curve (AUROC) with the following hyperparameters: learning rate, epochs, hidden dimensions, and number of features to be used for input (ranked according to the feature importance ranking). After hyperparameter tuning, we finalized training with the optimal combination of hyperparameters.

Model development was carried out using the previously mentioned TARNet algorithm. Conceptually, the model is a deep neural network that first learns a representation of the entire training sample. The model then branches off into a treatment and control layer. Each layer yields a probability-based prediction of the 28-day mortality for a patient, assuming they are part of the intervention group or the control group. The difference between these predictions can be defined as the individual treatment effect (ITE). Details of the TARNet methodology and comparisons with other algorithms can be found in the Supplementary Materials.

Model explainability was explored by calculating SHAP (Shapley Additive exPlanations) values and constructing three beeswarm plots based on the internal train set of AmsterdamUMCdb: (1) for mortality prediction assuming corticosteroids are administered, (2) for mortality prediction assuming corticosteroids are not administered, and (3) for the ITE. Evaluation was performed on the test set of AmsterdamUMCdb and MIMIC-IV. Only the observed outcomes could be compared with the predicted outcomes, as the counterfactual outcomes are, by definition, unobserved. Model performance was evaluated using the AUROC, Brier score, and a visual assessment of the calibration curves.

To assess covariate balance between control and intervention groups in both the internal (AmsterdamUMCdb) and external (MIMIC-IV) datasets, we computed the Wasserstein metric. The Wasserstein distance quantifies the cost of transforming one probability distribution into another. Lower values indicate greater similarity and overlap between distributions, while higher values suggest more pronounced differences. This metric was evaluated using the original datasets comprising 19 covariates as well as the latent representations obtained after processing through the TARNet deep learning model. We compared the resulting Wasserstein distances to those obtained using conventional propensity score matching (PSM), a widely used method for balancing covariate distributions between intervention and control groups.

### Classification of corticosteroid responders

After model development and evaluation, an ITE was calculated for all included patients of AmsterdamUMCdb. Patients with sepsis with a reduction of predicted mortality probability of >10% (ITE ≤ −10%) were classified as responders. Non-responders were defined as having an ITE between −10% and +10%. Patients with sepsis with an increase of >10% of predicted mortality (ITE ≥ +10%) were classified as adverse responders. Given that there is no literature for such cut-off values, we chose these based on clinical relevance: any physician would change their treatment plan if it meant a 10% change in survival rate for their patient. For visual inspection of the ITE predictions, a plot was drawn that ranked the ITEs from low to high for all included patients. The three groups, namely, corticosteroid responders, non-responders, and corticosteroid adverse responders were bootstrapped 1000 times to construct confidence intervals for all included AmsterdamUMCdb patients with sepsis.

## Results

### Cohort selection and baseline characteristics

Out of 23,106 admissions, 2920 were eligible according to the Sepsis-3 criteria. Of these, 1378 received therapeutic dosages of corticosteroids within 72 h of admission and were assigned to the treatment group, while the other 1542 were assigned to the control group. Table 1 presents the admission characteristics for each intervention group. Four demographic variables and 15 laboratory and physiological variables were extracted. The most deviating variable during the first 24 h of admission was extracted as specified in the methodology. Similar characteristics in both groups were sex, age, weight, height, pH, sodium, potassium, temperature, white blood cell count, glucose, and heart rate. Overall, the treatment group had a lower PaO_2_/FiO_2_ and bicarbonate values, and a higher percentage of ventilated patients, lactate, vasopressor dosage, creatinine, blood urea nitrogen, and C-reactive protein compared with the control group.

**Table 1:** Admission characteristics of the patients.

Characteristics	All admissions with sepsis (*n*=2920)	Treatment group (*n*=1378)	Control group (*n*=1542)
Male sex	1752 (60.0)	824 (59.8)	927 (60.1)
Age (years)	65 (55–75)	65 (55–75)	65 (55–75)
Weight (kg)	75 (65–85)	75 (65–85)	75 (65–85)
Height (cm)	175 (165–185)	175 (165–185)	175 (165–185)
28-day mortality	800 (27.4)	464 (33.7)	335 (21.7)
Variables measured within 24 h of admission			
Ventilated	2301 (78.8)	1217 (88.3)	1082 (70.2)
Lowest PaO_2_/FiO_2_-ratio	158.5 (100.0–230.0)	133.3 (90.0–197.5)	185.7 (116.7–260.0)
Lowest pH	7.3 (7.2–7.3)	7.2 (7.2–7.3)	7.3 (7.2–7.4)
Lowest bicarbonate (mmol/L)	19.0 (15.5–22.2)	17.5 (14.2–21.1)	20.1 (17.0–22.8)
Highest lactate (mmol/L)	2.4 (1.4–4.9)	3.0 (1.6–6.2)	1.9 (1.2–3.4)
Highest vasopressor dosage (norepinephrine equivalent in μg/(kg·min))	0.2 (0.0–0.8)	0.4 (0.2–1.2)	0.1 (0.0–0.4)
Highest creatinine (μmol/L)	112.0 (79.0–187.0)	131.0 (90.0–220.5)	101.0 (72.0–153.0)
Highest blood urea nitrogen (mmol/L)	9.8 (6.2–16.1)	11.3 (7.0–18.1)	8.6 (5.4–14.2)
Highest sodium (mmol/L)	142.0 (138.0–146.0)	142.0 (139.0–146.0)	142.0 (138.0–145.0)
Highest potassium (mmol/L)	4.5 (4.2–5.0)	4.7 (4.4–5.1)	4.4 (4.1–4.8)
Highest temperature (°C)	37.3 (36.7–38.0)	37.3 (36.7–38.0)	37.3 (36.7–38.0)
Highest C-reactive protein (mg/L)	145.0 (64.0–254.0)	158.0 (71.8–262.2)	137.0 (54.0–245.0)
Highest white blood cell count (×10^9^/L)	14.1 (9.3–21.0)	14.8 (9.1–22.4)	13.9 (9.4–19.9)
Highest glucose (mmol/L)	10.1 (8.3–12.5)	10.7 (8.8–13.4)	9.6 (7.9–11.7)
Median heart rate (beats/min)	90 (77–105)	92 (79–108)	88 (76–102)

Data presented as *n* (%) or median (interquartile range).

PaO_2_/FiO_2_: Partial pressure of oxygen /fraction of inspired oxygen ratio.

The external validation set of MIMIC-IV was comprised of 30,639 admissions that were included according to the Sepsis-3 criteria. Overall, the MIMIC-IV cohort of patients with sepsis had a notably lower severity of illness during the first 24 h of admission, indicated by a lower percentage of mechanical ventilation (78.8% *vs.* 12.2%). In-hospital mortality was also lower in the MIMIC-IV cohort (19.8% *vs.* 27.4%). Other characteristics were more similar. The complete characteristics admission table of the MIMIC-IV cohort can be found in the Supplementary Materials.

### Feature importance ranking and model evaluation metrics

The data were split into a training (*n*=2336) and test (*n*=584) set. The training set with 19 variables was then run through the model development pipeline. Feature importance ranking ordered all 19 variables in the following order: lactate, age, ventilation status, temperature, pH, blood urea nitrogen, weight, heart rate, bicarbonate, white blood cell count, creatinine, PaO_2_/FiO_2_, potassium, C-reactive protein, height, sex, sodium, glucose, and vasopressor dosage. The optimum number of features to choose was 11. Model explainability was visually assessed using SHAP beeswarm plots. For general mortality prediction, more weight was attributed to age, lactate, heart rate, and creatinine values. When assessing the explainability of the ITE estimation, which quantifies patient benefit from corticosteroid treatment, more weight was given to pH and body temperature. All SHAP beeswarm plots can be found in the Supplementary Materials.

**Figure 2:**
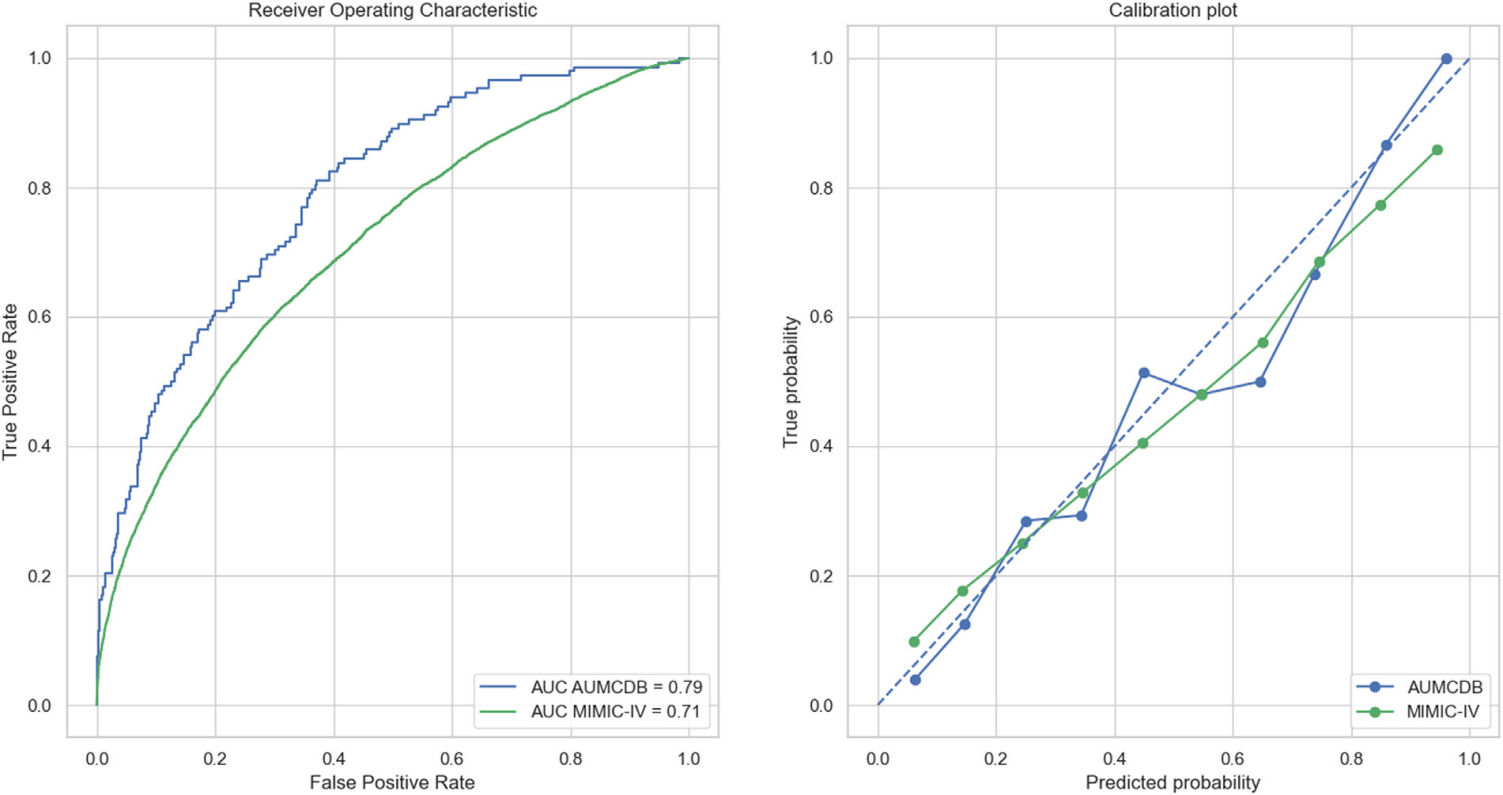
Receiver operating Characteristic curve (A) and calibration plot (B) of the internal validation set (AmsterdamUMCdb) and external validation set (MIMIC-IV). AUC: Area under the curve; AUMCDB: AmsterdamUMCdb; MIMIC-IV: Medical information mart for intensive care IV.

When evaluating the predictive performance, the TARNet model scored an AUC of 0.79 and a Brier score of 0.14 in the internal (AmsterdamUMCdb) test set. In external validation on the MIMIC-IV data set, an AUC of 0.71 and a Brier score of 0.14 were achieved. Calibration curves for both test sets were visually assessed and deemed adequate. The results are shown in Figure 2.

Besides TARNet, methods that have been applied within the causal modeling framework (specifically meta-learners) are logistic regression, XGBoost, and multilayer perceptron. They all performed similarly with respect to discrimination. All methods within different meta-learner frameworks (S-learner, T-learner, X-learner) and the conventional Propensity Score Method resulted in a similar internal AUC of 0.77–0.79 and an external AUC of 0.71–0.73. These results are similar to the predictive performance of the TARNet model. The complete overview of results for all models can be found in the Supplementary Materials.

When evaluating the covariate balance, for the AmsterdamUMCdb data set, the Wasserstein distance between the intervention and control group was 0.39. The comparator PSM model had a lower Wasserstein distance of 0.28 for the matched intervention and control groups. The latent representation created by the TARNet model had the lowest value of 3.6 × 10⁻⁷, indicating almost perfect covariate balance. For the external MIMIC-IV data set, the TARNet model also scored the lowest value (4.2 × 10⁻⁷). The covariate balance metrics can be found in Table 2.

**Table 2:** Wasserstein distance between the intervention and control group for each modeling technique.

Definition of groups	AmsterdamUMCdb (internal data set)	MIMIC-IV (external data set)
Original intervention and control group	0.39	0.29
Matched intervention and control group with PSM	0.28	0.33
Latent representation of intervention and control group with TARNet	3.6 × 10⁻⁷	4.2 × 10⁻⁷

Lower values indicate more similar distributions. A value of 0 represents two identical distributions. MIMIC-IV, medical information mart for intensive care IV; PSM, propensity score matching; TARNet, treatment agnostic representation network.

### Individual treatment effect estimations and subgroup characteristics

**Figure 3:**
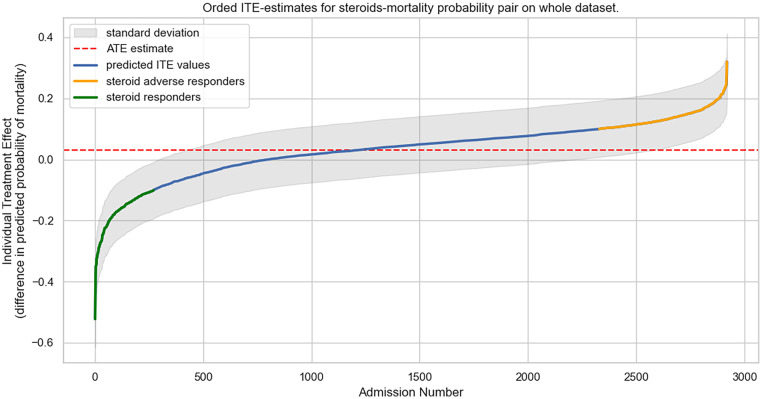
Ordered ITE estimates for corticosteroids-mortality probability pair on the whole dataset. The *x*-axis represents the 2930 admissions from the data set. The *y*-axis represents the ITE, showing the ITE per patient for corticosteroids. The ATE is defined as the mean of all ITEs. ATE: Average treatment effect; ITE: Individual treatment effect.

An ITE on 28-day mortality for corticosteroids was estimated for all included patients of the AmsterdamUMCdb data set. The ITEs were sorted and plotted in Figure 3. The average treatment effect (ATE) was defined as the mean of all ITEs, and was predicted to be 0.03. Defining the corticosteroid responders as those with an ITE of −10%, 245 admissions were identified. The majority of admissions were non-responders (*n*=2098), while 577 admissions were predicted to be corticosteroid adverse responders.

Characteristics were inspected among the groups of corticosteroid responders, non-responders, and adverse responders. Bootstrapping was performed to retrieve a 95% confidence interval. Mean heart rate, lactate, blood urea nitrogen, creatinine, potassium, glucose, and white blood cell count were significantly higher in the corticosteroid responders group than in the non-responders and adverse responders groups. Characteristics that were significantly lower in the corticosteroid responders group were arterial pH, bicarbonate, PaO_2_/FiO_2_, C-reactive protein, and temperature. Table 3 shows the characteristics for each group.

**Table 3:** Characteristics per ITE group.

Characteristics	Non-responders (*n*=2098)	Responders (*n*=245)	Adverse responders (*n*=577)
Male sex (%)	59 (57 – 61)	65 (59 – 71)	60 (56 – 64)
Age (years)	60.8 (60.1 – 61.5)	67.2 (65.5 – 68.9)	65.8 (64.5 – 67.2)
Weight (kg)	79.2 (78.5 – 79.8)	78.59 (76.88 – 80.31)	74.13 (73.08 – 75.19)
Height (cm)	175.0 (174.6 – 175.4)	175.0 (173.9 – 176.1)	173.5 (172.8 – 174.2)
Ventilation status (%)	73 (71 – 75)	93 (90 – 96)	93 (91 – 95)
PaO_2_/FiO_2_	192.9 (188.5 – 197.6)	133.5 (125.0 – 142.2)	156.5 (150.6 – 162.9)
Vasopressor dosage (μg/(kg·min)	2.5 (1.1 – 5.0)	2.8 (2.3 – 3.4)	1.2 (1.0 – 1.5)
Arterial pH	7.26 (7.26 – 7.27)	7.04 (7.03 – 7.06)	7.33 (7.32 – 7.34)
Heart rate (beats/min)	90.5 (89.7 – 91.3)	98.6 (95.9 – 101.6)	90.3 (88.8 – 92.0)
Blood urea nitrogen (mmol/L)	11.8 (11.4 – 12.2)	18.4 (17.0 – 20.0)	12.6 (11.9 – 13.3)
Creatinine (μmol/L)	155.8 (150.4 – 162.5)	274.8 (245.9 – 306.1)	123.5 (117.4 – 130.1)
C-Reactive protein (mmol/L)	161.4 (157.0 – 166.0)	137.8 (124.6 – 150.7)	170.5 (162.6 – 179.5)
Glucose (mmol/L)	10.8 (10.6 – 11.0)	14.0 (13.2 – 14.9)	10.9 (10.6 – 11.3)
Lactate (mmol/L)	3.4 (3.3 – 3.6)	7.9 (7.3 – 8.6)	3.1 (2.9 – 3.2)
White blood cell count (×10^9^/L)	16.2 (15.8 – 16.6)	22.2 (20.5 – 23.9)	12.6 (11.9 – 13.2)
Potassium (mmol/L)	4.7 (4.6 – 4.7)	5.2 (5.1 – 5.4)	4.5 (4.5 – 4.6)
Sodium (mmol/L)	141.8 (141.5 – 142.0)	143.6 (142.7 – 144.5)	143.2 (142.7 – 143.7)
Temperature (°C)	37.3 (37.3 – 37.4)	36.0 (35.9 – 36.2)	37.9 (37.8 – 38.0)
Bicarbonate (mmol/L)	18.6 (18.4 – 18.8)	13.0 (12.4 – 13.7)	21.9 (21.5 – 22.3)

Data are presented as means (95% confidence intervals).

PaO_2_/FiO_2_: Partial pressure of oxygen /fraction of inspired oxygen ratio; ITE, individual treatment effect.

## Discussion

To the best of our knowledge, this is the first study to use causal deep learning to identify patients with sepsis who respond to corticosteroid treatment. We built a pipeline for two large-scale, highly granular electronic health records based databases. Nineteen clinically relevant variables were selected and processed through this pipeline for one database (AmsterdamUMCdb). These features were selected based on the domain knowledge of intensivists and their usage in existing mortality prediction models.^[16^^,^^17]^ From a physiological standpoint, patients with sepsis are often severely ill, where they have a compromised hemodynamic circulation (creatinine, vasopressor dosage, heart rate, blood urea nitrogen, lactate, potassium, sodium, bicarbonate, pH) due to severe inflammation (as indicated by C-reactive protein, white blood cell count, temperature). Their course in the ICU is also often determined by their physical reserves (sex, age, weight, height, ventilation status). While the Missing Completely at Random condition cannot be guaranteed, the low occurrences of the features’ missingness (0.3%–16.1%) mitigated the potential bias that was introduced during imputation (Supplementary Materials). The variables were then used to train a deep neural network; this involved extensive hyperparameter tuning. To further validate the model, external validation on a data set with a different case mix of ICU patients was carried out (MIMIC-IV). This finally yielded a model capable of computationally estimating the treatment effect of corticosteroids for individual patients with sepsis using routinely measured variables.

Few data-driven approaches have been applied for optimal treatment strategies, specifically in the context of corticosteroid treatment for sepsis. Pirracchio et al.^[22]^ found a favorable outcome for machine learning based, individualized corticosteroid therapies when compared with a generalized treatment strategy. Offline reinforcement learning has also been applied to learn optimal treatment strategies for patients with sepsis, including the usage of corticosteroids, which suggests a theoretical advantage for the artificial intelligence based algorithm.^[23^^,^^24]^ However, these methods were primarily used to assess their performance compared with clinicians or a baseline strategy; they are not applied to more accurately characterize patients that respond to corticosteroids, which is a key element to aid clinicians in making therapeutic decisions. Furthermore, it is highly preferable to have all used databases and code bases be publicly available, facilitating transparency, trustworthiness, and adoption.

A strength of our approach is the application of a TARNet, a state-of-the-art causal inference method for estimating the individual treatment effect. Fundamentally, it is a supervised learning prediction model that is used to predict the counterfactual: what would the probability of mortality be had the patient received a different treatment? Previous methodologies have been applied to these issues, incorporating designs such as G-estimation, regression, and causal forests.^[25]^ However, when comparing these methods, TARNet outperforms most other algorithms in simulations, as described by Shalit et al.^[21]^ Furthermore, it uses a conceptually intuitive method of calculating the counterfactual; by using deep neural networks, it learns a representation of all patients and then splits them into a treatment and control group for further modeling. It combines elements from the transparent method of G-estimation and improved predictions of deep neural networks, and can easily include methods for variance bounding with Integral Probability Metrics between treated and control distributions. Consequently, the TARNet model also adjusted for the more unwell treatment group to mitigate confounding by indication. When comparing the covariate balance between the original treatment and control groups, the TARNet model significantly reduced the value to near 0 for both the internal (3.6 × 10⁻⁷) and external (4.2 × 10⁻⁷) data set, indicating that the deep learning model created equal control and intervention groups with regard to the included variables. More details regarding the TARNet methodology are presented in the Supplementary Materials. By applying TARNet on a large, highly granular, public ICU database, we were able to make individual estimates of the predicted 28-day mortality. When validating the performance on the observed outcomes, a solid discrimination performance was achieved on both the internal and the external validation test set (internal AUC AmsterdamUMCdb=0.79, external AUC MIMIC-IV=0.71). The model was also considered well calibrated based on the calibration curves.

The model showed that corticosteroid responders were those with severe metabolic acidosis and impaired circulation. Comparison of the responders with the non-responders, were the former were defined as having a 10% predicted reduction in 28-day mortality, revealed significant differences with respect to pH, bicarbonate, lactate, creatinine, blood urea nitrogen, potassium, and heart rate. These findings fit with previous trials where a mortality benefit for corticosteroids was found in Annane et al.,^[26]^ which included patients with refractory septic shock. The same benefit was not found in the CORTICUS trial, which included fewer unwell patients with sepsis.^[27]^ These results are also in line with the latest guidelines, which suggest the use of corticosteroids in adult patients with septic shock, the most unwell subgroup of patients with sepsis.^[9]^

Furthermore, comparison of the metabolic profiles of the responders and the actual treatment group of the AmsterdamUMCdb database also reveals some marked differences. The treatment group typically included patients with high vasopressor dosages, but not necessarily signs of impaired circulation based on biomarkers (e.g., lower pH, lower bicarbonate, and higher lactate). This implies that the physicians of the Amsterdam-based hospital gave considerable weight to vasopressor dosage in the decision to start corticosteroid therapy, even though initial metabolic and circulation biomarkers might be more predictive of their being corticosteroid responders. This discrepancy between the physicians’ decisions to initiate corticosteroid therapy and the model’s predictions of being a corticosteroid responder implies that conventional clinical reasoning is not ideal for complex clinical decision-making for long-term outcomes. Rather, it may be necessary to combine these forms of reasoning with the use of computational systems that can process a multitude of variables for optimal-decision making. Previous literature also supports that the treatment of patients with septic shock should be based on estimated individual treatment effects as derived from machine learning models.^[22]^

Finally, estimation of the ATE of corticosteroids over the whole group revealed it to be negligible (ATE=0.03). This resulted from the treatment effect for the responders (*n*=245) being canceled out by the treatment effect for the majority of non-responders (*n*=2087) and “adverse responders,” i.e., those who were predicted to be harmed by corticosteroid treatment (*n*=579). This further implies that a clinical trial with these patients would have yielded negative results because of the average effect for the responders, non-responders, and adverse responders. However, when setting inclusion criteria for a prospective trial to more closely match the group of either responders or adverse responders, a well-powered, targeted clinical trial may be feasible. This would validate the study even further, given its inherent limitation of using observational data: it cannot be assumed that all confounders are observed and accounted for. Validating the counterfactual results is also not possible, given that they are, by definition, not observed in the dataset.

However, even though conventional randomized clinical trials are the gold standard for inferring causality, they are often constrained by ethical, logistical, or financial concerns. Consequently, researchers have found ways to imply causality by using large amounts of observational data with general assumptions based on medical knowledge. For instance, by combining medical knowledge about tobacco and large observational data, strong conclusions could be made that smoking is a causal factor in various cancers.^[28]^ Similarly, by assuming that corticosteroids are beneficial for the most unwell patients, as inferred from previous trials,^[26^^,^^27]^ clinicians can leverage the findings from these large datasets to more accurately define which patients with sepsis can be classified as steroid responders or steroid adverse responders. While our work requires prospective evaluation and implementation testing in different healthcare settings, the findings offer the potential for a reduction in sepsis mortality, which would have a significant clinical impact. Given that new treatments for sepsis have not been forthcoming in recent times, implementing personalized decision support for improved healthcare outcomes is a worthwhile approach for future investigations.

## CRediT authorship contribution statement

**Ameet Jagesar:** Writing – review & editing, Writing – original draft, Visualization, Methodology, Formal analysis, Data curation, Conceptualization. **Louk Smalbil:** Writing – review & editing, Methodology, Formal analysis, Conceptualization. **Etienne Galea:** Data curation. **Tristan Struja:** Data curation. **Tariq Dam:** Writing – review & editing. **Paul Hilders:** Writing – review & editing. **Martijn Otten:** Visualization, Writing – review & editing. **Laurens Biesheuvel:** Visualization, Writing – review & editing. **Armand Girbes:** Supervision, Resources. **Patrick Thoral:** Resources, Data curation. **Mark Hoogendoorn:** Writing – review & editing, Supervision. **Paul Elbers:** Writing – review & editing, Supervision, Resources.

## Appendix Supplementary materials

Image, application 1

Image, application 2
