# Supplementary material for: Causal deep learning to personalize medicine: Which intensive care patients with sepsis will benefit from corticosteroid therapy?
Source: J Intensive Med. 2025 Sep 23;6(1):61–8. doi: 10.1016/j.jointm.2025.07.002 (PMC12925864; doi:10.1016/j.jointm.2025.07.002)
Supplement: Supplementary file 2 [file mmc2.doc]

# Causal deep learning for identification of steroid responders in sepsis - supplementary materials

## Data preprocessing

## Imputation method per variable

#### AmsterdamUMCdb:

| Variable | Percentage missing | Imputation method | Imputation value |
| --- | --- | --- | --- |
| Sex | 0.0% | Not applicable | Not applicable |
| Age (years) | 0.0% | Not applicable | Not applicable |
| Weight (kg) | 3.9% | Median | 75.0 |
| Height (cm) | 7.5% | Median | 175.0 |
| Mechanical ventilation status | 28.2% | Normal value | 0 (no registered ventilation status was assumed to be not ventilated) |
| P/F ratio | 11.8% | Normal value | 400 |
| Arterial pH | 0.8% | Median | 7.3 |
| Bicarbonate | 0.6% | Median | 19.0 |
| Lactate | 16.1% | Median | 2.4 |
| Vasopressor dosage (gamma) | 44.8% | Normal value | 0 (no registered administration of vasopressors are assumed to be not given) |
| Creatinine | 1.8% | Median | 112.0 |
| Blood urea nitrogen | 7.5% | Median | 9.9 |
| Sodium | 0.3% | Median | 142.0 |
| Potassium | 0.3% | Median | 4.5 |
| Temperature | 0.5% | Median | 37.3 |
| C-reactive protein | 26.4% | Median | 147.0 |
| White blood cell count | 1.4% | Median | 14.1 |
| Glucose | 0.3% | Median | 10.1 |
| Heart rate | 0.0% | Not applicable | Not applicable |

#### MIMIC-IV:

| Variable | Percentage missing | Imputation method | Imputation value |
| --- | --- | --- | --- |
| Sex | 0.0% | Not applicable | Not applicable |
| Age (years) | 0.0% | Not applicable | Not applicable |
| Weight (kg) | 1.7% | Median | 75.0 |
| Height (cm) | 45.8% | Median | 175.0 |
| Mechanical ventilation status | 0.0% | Not applicable | Not applicable |
| P/F ratio | 49.9% | Normal value | 400 |
| Arterial pH | 25.1% | Median | 7.3 |
| Bicarbonate | 0.2% | Median | 19.0 |
| Lactate | 33.7% | Median | 2.4 |
| Vasopressor dosage (gamma) | 50.8% | Normal value | 0 (no registered administration of vasopressors are assumed to be not given) |
| Creatinine | 0.1% | Median | 112.0 |
| Blood urea nitrogen | 0.1% | Median | 9.9 |
| Sodium | 0.2% | Median | 142.0 |
| Potassium | 0.2% | Median | 4.5 |
| Temperature | 4.0% | Median | 37.3 |
| C-reactive protein | 98.0% | Median | 147.0 |
| White blood cell count | 0.2% | Median | 14.1 |
| Glucose | 0.6% | Median | 10.1 |
| Heart rate | 0.0% | Median | 90.0 |

## External dataset admission characteristics (MIMIC-IV)

| Characteristic | all sepsis admissions (n = 30639) | treatment group (n = 876) | control group (n = 29763) |
| --- | --- | --- | --- |
| Male sex (percentage) | 58,0% | 55,5% | 58,0% |
| Age (years) | 68 (57 - 78) | 66 (57 - 74) | 68 (57 - 79) |
| Weight (kg) | 79 (66 - 94) | 78 (65 - 95) | 79 (66 - 94) |
| Height (cm) | 170 (163 - 178) | 168 (160 - 178) | 170 (163 - 178) |
| 28-day mortality | 19,8% | 27,1% | 19,6% |
| Variables measured within 24 hours of admission |  |  |  |
| Ventilated (percentage) | 12,2% | 13,7% | 12,2% |
| Lowest PF-ratio | 400.0 (178.3 - 400.0) | 290.8 (96.0 - 400.0) | 400.0 (182.0 - 400.0) |
| Lowest pH | 7.3 (7.3 - 7.4) | 7.3 (7.2 - 7.4) | 7.3 (7.3 - 7.4) |
| Lowest bicarbonate (mmol/L) | 22.0 (18.0 - 24.0) | 21.0 (17.0 - 24.0) | 22.0 (18.0 - 24.0) |
| Highest lactate (mmol/L) | 2.3 (1.5 - 3.6) | 2.5 (1.6 - 4.4) | 2.3 (1.5 - 3.5) |
| Highest vasopressor dosage (norepinephrine equivalent in gamma) | 0.0 (0.0 - 0.2) | 0.0 (0.0 - 0.3) | 0.0 (0.0 - 0.2) |
| Highest creatinine (micromol/L) | 106.1 (70.7 - 176.8) | 106.1 (79.6 - 176.8) | 106.1 (70.7 - 176.8) |
| Highest blood urea nitrogen (mmol/L) | 8.9 (5.7 - 15.0) | 10.0 (6.4 - 15.4) | 8.9 (5.7 - 15.0) |
| Highest sodium (mmol/L) | 140.0 (137.0 - 143.0) | 140.0 (137.0 - 143.0) | 140.0 (137.0 - 142.0) |
| Highest potassium (mmol/L) | 4.5 (4.1 - 5.0) | 4.7 (4.3 - 5.3) | 4.5 (4.1 - 5.0) |
| Highest temperature (C) | 37.3 (36.9 - 37.9) | 37.3 (37.0 - 37.8) | 37.3 (36.9 - 37.9) |
| Highest C-reactive protein (mg/L) | 90.0 (34.5 - 175.8) | 88.6 (40.0 - 189.1) | 90.0 (33.7 - 175.1) |
| Highest white blood cell count (x10^9/L) | 13.5 (9.6 - 18.6) | 14.1 (9.2 - 19.4) | 13.5 (9.6 - 18.6) |
| Highest glucose (mmol/L) | 8.0 (6.5 - 10.9) | 10.4 (8.0 - 14.6) | 8.0 (6.5 - 10.8) |
| Median heart rate (beats/min) | 86 (76 - 98) | 87 (75 - 101) | 86 (76 - 98) |

### Definition of high-dose steroids

High-dose steroids were defined as follows:

- Hydrocortisone: > 200mg / day
- Prednisone: > 50mg / day
- Methylprednisolone: > 40mg / day
- Dexamethasone: > 8mg / day

Daenen *et al.* (2023), Table 1

## Treatment Agnostic Representation Network (TARNet) methodology

We used the causality framework of Rubin-Neyman (Rubin, 2005). For an admission with characteristics $x \backslash in X$ and a treatment $t \backslash in (0,1)$, there are two potential outcomes: $Y\_\{0\}$ and $Y\_\{1\}$. When using real-world data, only one outcome is observed for each admission depending on the given treatment: if $t = 0$ then we observe outcome $Y\_\{0\}$ and if $t = 1$ we observe outcome $Y\_\{1\}$. We aimed to estimate the unobserved (counterfactual) outcome with prediction modeling. The goal was to estimate the individual treatment effect (ITE):

$$\tau(x):=\boldsymbol{E}[Y1-Y0\mid x]$$

For such modeling purposes in a causality framework, the strong ignorability assumption is required. Meaning there are no ‘hidden confounders’ in the data set: $Y \{\backslash perp \backslash!\backslash!\backslash! \backslash perp\} t|x$ and $0 < p(t = 1|x) < 1$ for all $x$. This assumption cannot be validated based on mere data and requires domain knowledge.

We trained a TARNet model $m$ for estimating the observed and unobserved outcomes, which we can use for calculating the ITE:

*τ*(*x*) := **E**[Y_1_​−Y_0_​∣*x*] = f_1_​(*x*)−f_0_​(*x*)

The difference of TARNet with standard supervised machine learning approaches is the bounding of variance using an Integral Probabilistic Metric (IPM) for the distance between the control and treated distributions: $p(x|t = 0)$ and $p(x|t = 1)$ (Shalit *et al.*, 2017). This leads to a form of bias-variance trade-off that outperforms state-of-the-art algorithms in simulation experiments. For more in-depth information, we refer to the original paper.


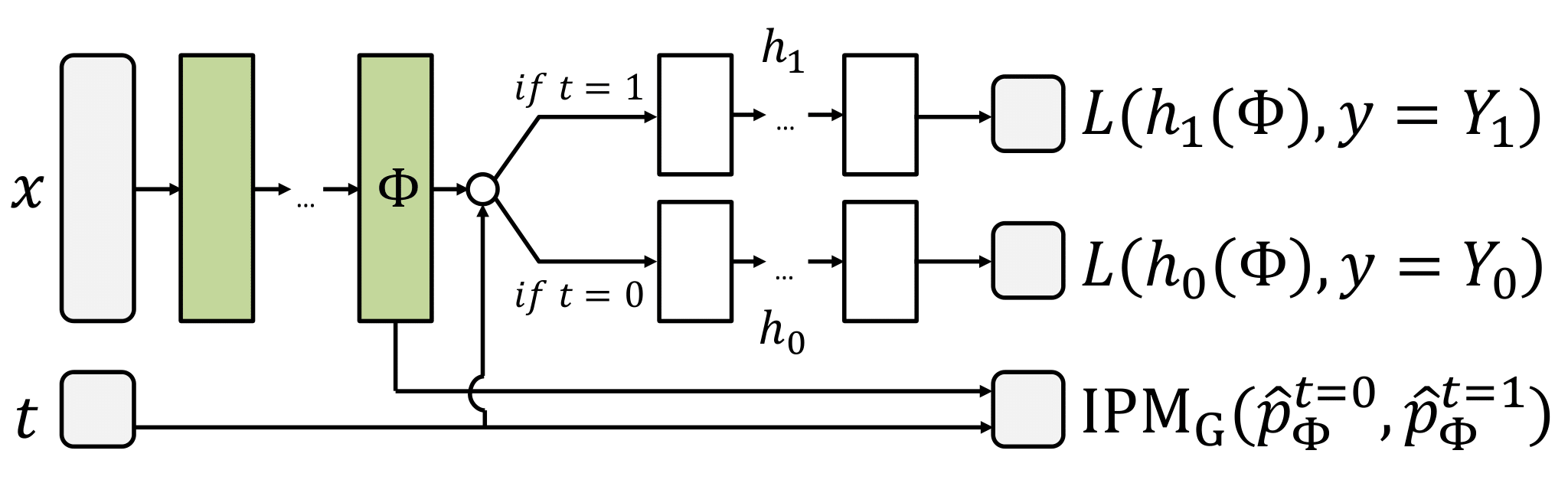


The TARNet neural network architecture. ϕ = learned representation of features $x$, IPM = integral probability metric, L = loss function. Image from Shalit *et al.* (2017).

## Feature importance

Using a logistic regression model for recursive feature elimination with cross-validation, the combination of input features were ranked as follows:

| Rank | Feature |
| --- | --- |
| 0 | Lactate |
| 1 | Age |
| 2 | Ventilation status |
| 3 | Temperature |
| 4 | Arterial pH |
| 5 | Blood urea nitrogen |
| 6 | Weight |
| 7 | Heart rate |
| 8 | Bicarbonate |
| 9 | White blood cell count |
| 10 | Creatinine |
| 11 | P/F ratio |
| 12 | Potassium |
| 13 | C-Reactive protein |
| 14 | Height |
| 15 | Sex |
| 16 | Sodium |
| 17 | Glucose |
| 18 | Vasopressor dosage |

## Hyperparameter tuning results

Using the Optuna library, hyperparameter tuning was performed on the train set (Akiba *et al.*, 2019). 20% of the train set was used for evaluation of hyperparameters using AUC of the observed outcome as metric. The following hyperparameters were optimised:

- Number of features to include, range of 3 to 19 -> optimal hyperparameter: 11
- Learning rate, range of 1e-6 to 1e-1 -> optimal hyperparameter: 5.3e-06
- Epochs, range of 3 to 20000 -> optimal hyperparameter: 11197
- Hidden dimensions, range of 4 to 128 -> optimal hyperparameter: 108

## Performance of all developed models

Using meta-learners and three different classifiers (Logistic Regression, XGBoost and Multilayer Perceptron), we achieved similar discrimination results compared with TARNet on the observed outcomes. Given similar performance metrics for observed outcomes, TARNet is the preferred choice given its improved prediction of ITE in simulated benchmarks.

The S-learner tends to bias the treatment effect towards zero, because it treats the treatment as a predictor that might be regularized down. T- and X-learners learn two different models (one for the treatment group and one for the control group) that causes more data sparcity to learn from.

| Meta-learner | Algorithm | AUC internal test set (AumsterdamUMCdb) | AUC external test set (MIMIC-IV) |
| --- | --- | --- | --- |
| S-learner | Logistic Regression | 0.78 | 0.72 |
| S-learner | XGBoost | 0.8 | 0.73 |
| S-learner | MLP | 0.79 | 0.72 |
| T-learner | Logistic Regression | 0.79 | 0.71 |
| T-learner | XGBoost | 0.78 | 0.72 |
| T-learner | MLP | 0.79 | 0.72 |
| X-learner | Logistic Regression | 0.79 | 0.71 |
| X-learner | XGBoost | 0.77 | 0.71 |
| X-learner | MLP | 0.79 | 0.72 |

## Shapley beeswarm plot of train set

## SHAP values for mortality prediction given t = 1 (steroids administered)

##
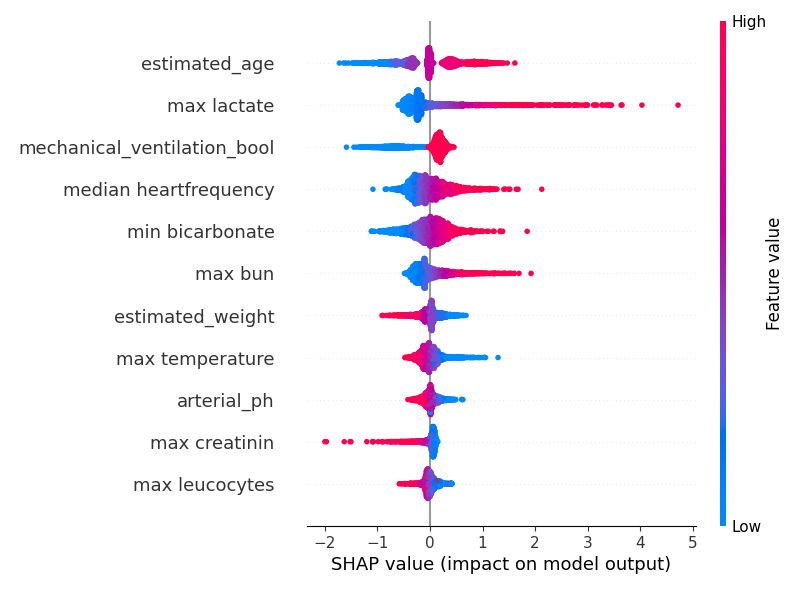


## SHAP values for mortality prediction given t = 0 (no steroids administered)

##
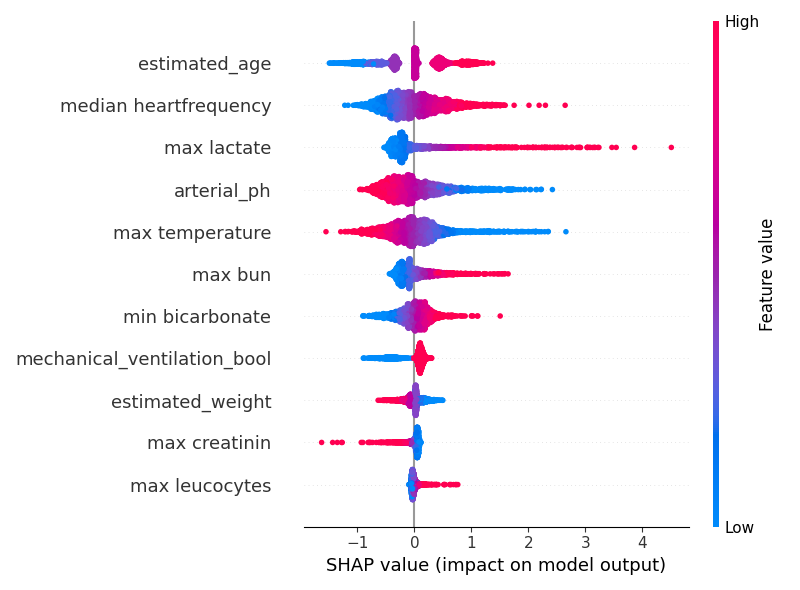


## SHAP values for individual treatment effect: (estimated mortality for t = 1) – (estimated mortality for t = 0)

##
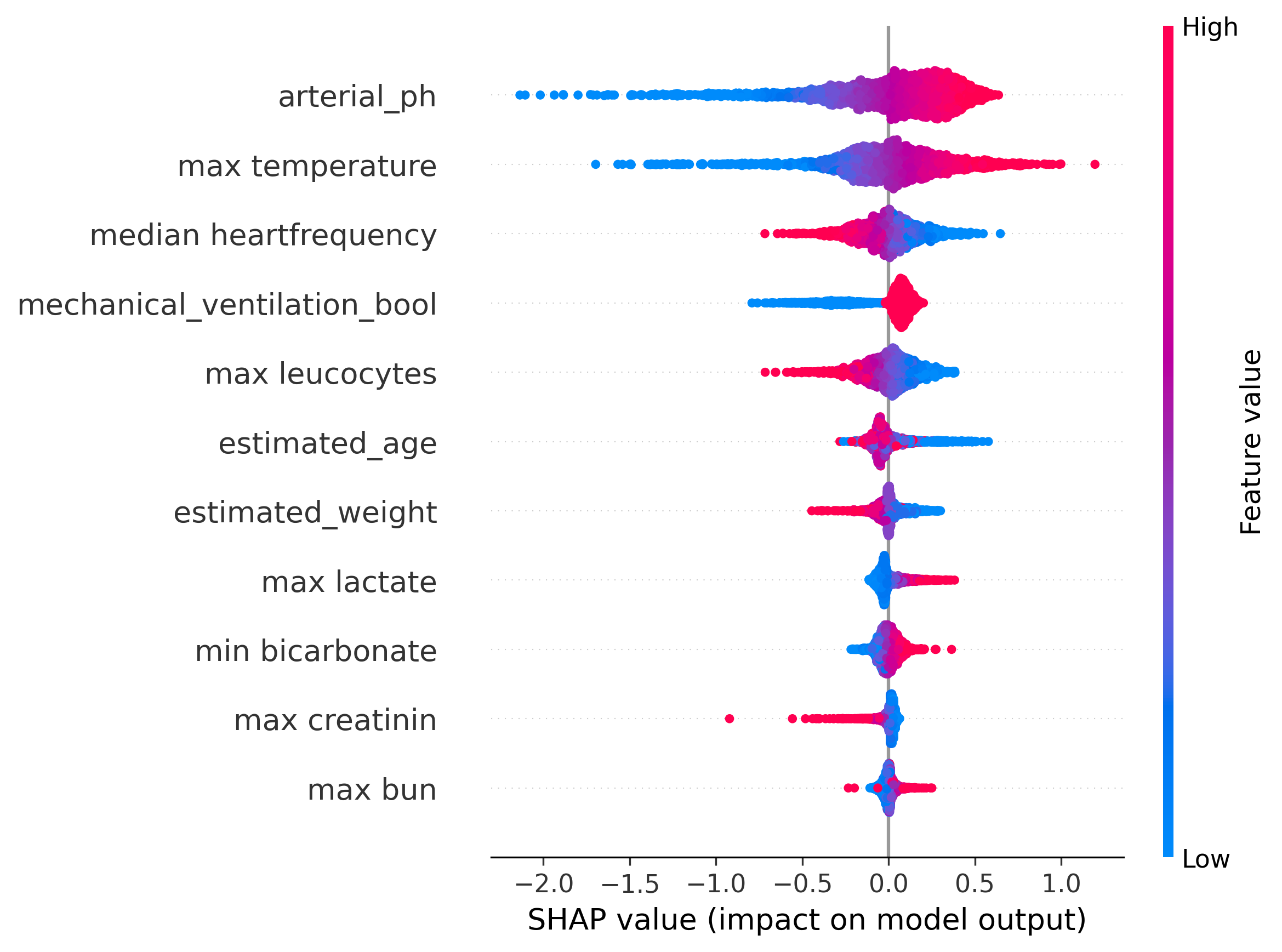


## References

Akiba, T., Sano, S., Yanase, T., Ohta, T., & Koyama, M. (2019). Optuna: A Next-generation Hyperparameter Optimization Framework. *Proceedings of the 25th ACM SIGKDD International Conference on Knowledge Discovery & Data Mining*, 2623–2631. [10.1145/3292500.3330701](https://doi.org/10.1145/3292500.3330701)

Daenen, K., Huijben, J. A., Boyd, A., Bos, L. D. J., Stoof, S. C. M., van Willigen, H., Gommers, D. A. M. P. J., Moeniralam, H. S., den Uil, C. A., Juffermans, N. P., Kant, M., Valkenburg, A. J., Pillay, J., van Meenen, D. M. P., Paulus, F., Schultz, M. J., Dalm, V. A. S. H., van Gorp, E. C. M., Schinkel, J., & Endeman, H. (2023). Optimal Dosing and Timing of High-Dose Corticosteroid Therapy in Hospitalized Patients With COVID-19: Study Protocol for a Retrospective Observational Multicenter Study (SELECT). *JMIR Research Protocols*, *12*, e48183. [10.2196/48183](https://doi.org/10.2196/48183)

Rubin, D. B. (2005). Causal Inference Using Potential Outcomes: Design, Modeling, Decisions. *Journal of the American Statistical Association*, *100*(469), 322–331. [10.1198/016214504000001880](https://doi.org/10.1198/016214504000001880)

Shalit, U., Johansson, F. D., & Sontag, D. (2017). Estimating Individual Treatment Effect: Generalization Bounds and Algorithms. *Proceedings of the 34th International Conference on Machine Learning*, 3076–3085. <https://proceedings.mlr.press/v70/shalit17a.html>
